# Fifteen years’ experience of radiation therapy for resected advanced heterotopic ossification following motor vehicle accidents: outcome and side effects

**DOI:** 10.1186/s43046-022-00149-w

**Published:** 2022-11-21

**Authors:** Reham Mohamed, Asif Iqbal, Abosaleh Abosaleh Elawadi

**Affiliations:** 1 National Cancer Institute, Radiotherapy and Nuclear Medicine Department, Cairo University, Cairo, Egypt; 2 Radiation Oncology Department, King Fahad Medical City, Riyadh, Saudi Arabia; 3 Medical Physics Department Riyadh, King Fahad Medical City, Riyadh, Saudi Arabia; 4 Faculty of Medicine, Clinical Oncology and Nuclear Medicine Department, Mansoura University, Mansoura, Egypt

**Keywords:** Heterotopic, Ossification, Radiotherapy, Hip, Elbow

## Abstract

**Background:**

Surgical resection is the primary treatment for advanced-stage heterotopic ossification (HO), with a high incidence of local recurrence reaching up to 50%. Postoperative radiotherapy (PORT) and indomethacin are commonly used prophylactic strategies following surgery. The study aims to assess the safety and effectiveness of PORT in advanced-stage HO patients having motor vehicle accidents (MVA).

**Methods:**

Medical records of patients having HO following MVA between 2006 and 2021 were retrospectively reviewed. Thirty-nine patients with advanced disease (35 had hip HO and 4 had elbow HO) were included in the study.

**Results:**

Excision of HO with joint preservation was performed for 82% of patients, while 18% had a joint replacement. Seven to 8 Gy radiation was given to all patients within 3 days postoperatively. A ninty seven percent of patients regained partially the movement range. The mean follow-up time was 74 months. Six patients had treatment failure, with only one having a recurrence of HO. The 8-year treatment failure-free rate (8-y TFFR) was 79.3±9%, and the 5-year HO failure-free rate (5y-HOFFR) was 97.2±3%. Acute side effects were experienced in 13% of patients but resolved without any consequences. Despite the relatively long follow-up time, we did not report any absolute infertility or secondary malignancies related to the radiation. The testicular mean calculated dose was 33±44 cGy, and the mean measured dose was 58±40 cGy. Of the 35 patients who received radiation to the pelvis, 26 were married, and all did not experience infertility post-treatment.

**Conclusion:**

PORT proved an effective and safe treatment for advanced-stage HO disease. The treatment failure is mainly related to surgical difficulties due to advanced disease. Treatment using a 3-dimensional or intensity-modulated radiation therapy is not associated with serious side effects like second malignancy or absolute infertility.

## Background

HO is a well-known benign condition characterized by abnormal bone formation around the joints limiting the range of movements (ROM) and affecting the quality of life [1]. Motor vehicle accidents (MVA) are the leading cause of HO nowadays. The clinically significant HO among the seriously fractured and dislocated joints was 23% and even up to 37% in patients with serious brain and/or spinal cord injuries [2–4]. The hip is the most commonly affected joint, followed by the elbow joint. The extent of such disease is graded by Brooker’s classification for the hip joints [5], while Hastings and Graham’s classification is used for the elbow joints [6].

Surgical resection is the optimal approach to regain functional outcomes with a high possibility of recurrence following resection. Successful surgical treatment of HO warrants gentle handling of the tissues, avoidance of excess bleeding, and good hemostasis. Postoperative hematoma and infection are the leading causes of surgical failure. Early postoperative prophylaxis is preferable as the tendency for HO recurrence increases with subsequent surgeries by nearly four-folds. The limb-threatening complications of multiple surgical resections like nerve entrapments justify the need for early prophylaxis [7–9]. The widely used methods for preventing HO re-formation include PORT within 4 days and/or indomethacin therapy by a dose of 75 to 100 mg/day for 2–6 weeks [10, 11]. Bleeding is a well-known side effect of indomethacin that could intervene with successful surgeries.

On the other hand, surgeons blame the PORT for the higher rate of postoperative infection and surgical failure. Studies showed contradictory results regarding the beneficial effect of radiation therapy and its complications; however, most published data was for patients treated with old primitive radiotherapy techniques [12, 13]. Apart from its rarity, infertility and second malignancy are the most serious expected complications for radiotherapy (RT). Using a 3-dimensional conformal radiation therapy (3D-CRT) or intensity-modulated radiotherapy (IMRT) can reduce infertility by keeping the radiation to the testis and ovaries within the acceptable tolerance doses. Proper shielding and avoidance of -radiation beam to pass through- organs at risk (OAR) are essential maneuvers to achieve that goal [14–16].

### Aim of the study

The most advanced-stage HO patients are referred to our hospital as a specialized medical city affiliated with the Ministry of Health (MOH). This study aims to present the outcome and complications of treating advanced-stage HO patients by adjuvant PORT using modern radiation therapy techniques like 3DCRT and IMRT.

## Methods

**Fig. 1:**
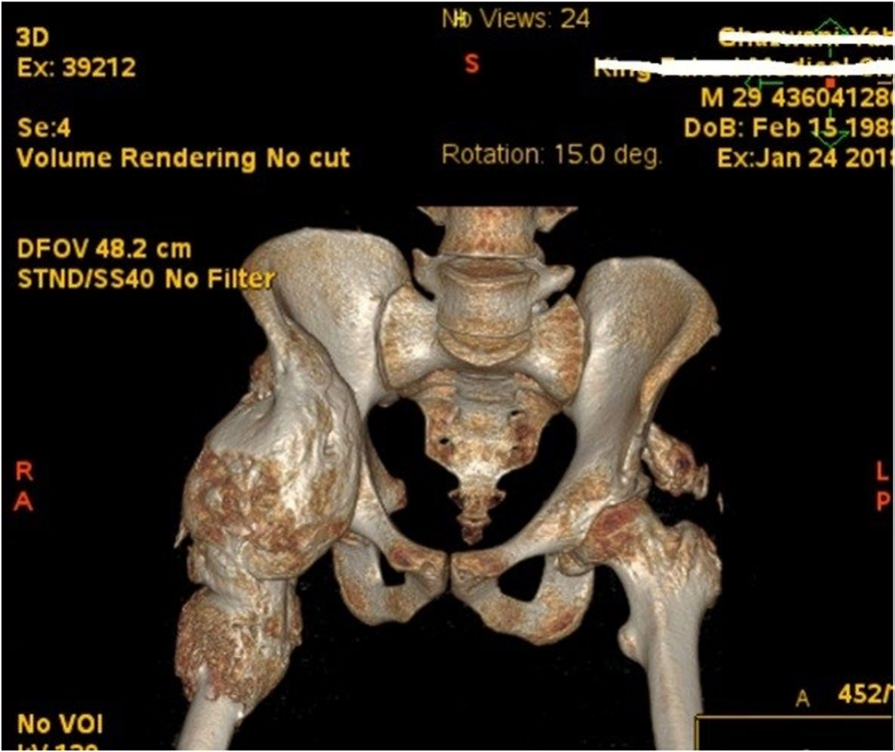
Patient with heterotopic ossification following brain injury. (3D osseous CT showing G2 Lt hip heterotopic ossification and G4 RT hip heterotopic ossification for a patient with long-standing bed bound stay following brain injury)

The HO patients presented to our hospital from 2006 to 2021 were retrospectively studied after the institutional review board (IRB) approval. Medical records of hospital information system (HIS) used for data collection. Patients with a history of MVA presented with G4 Brooker’s classification for hip joint or Class IIIc Hastings and Graham classification for the elbow joint were included in this study (Table 1 and Fig. 1). We excluded patients with early-stage disease, patients with missing data, and patients with causes rather than MVA.

**Table 1:** Heterotopic ossification staging

Brookers’ classification of HO of the hip joint		Hastings and Graham’s classification	
Grade	Description	Class	Description
**1**	Bone islands in the soft tissue around the hip	***Class I***	*HO without functional limitation*
**2**	Exophytes in the pelvis or proximal end of the femur with at least 1 cm between opposing bone surfaces	***Class II*** ***IIa*** ***IIb*** ***IIc***	*HO with functional limitation (limited ROM)* *Flexion/extension limitation* *Pronation/supination limitation* *Both A and B*
**3**	Exophytes in the pelvis or proximal end of the femur with less than 1 cm between opposing bone surfaces	***Class III*** ***IIIa*** ***IIIb*** ***IIIc***	*HO with ankylosis* *Flexion/extension limitation* *Pronation/supination limitation* *Both A and B*
**4**	Bony ankylosis between proximal femur and pelvis

The standard protocol at our hospital is to treat advanced-stage HO patients with surgery followed by radiotherapy within 24 h postoperatively.

### Surgical details

The surgical approach is determined by the location of HO, which is then exposed with an electric cautery to reduce bleeding. The covering muscles are released with respect and caution for the vessels, nerves, and the joint capsule. In contrast to oncological resection, HO does not need to have an extensive one with targeting only ossification-causing limitation of mobility, vascular, or nerve compression. Adequate drainage is performed to control hemorrhage during the first week postoperatively. The mobilization and physiotherapy are recommended with caution at least 1-week post-resection. The fracture below the resection line at the implantation base of HO represents the main risk during physiotherapy and mobilization. This risk is higher in our cohort of studied patients with joint ankylosis, significantly if the delayed correction exceeds 2 years post-trauma due to bone demineralization. Rehabilitation is then escalated 2 weeks post-surgical resection once the sutures had been removed.

### Radiotherapy details

**Fig. 2:**
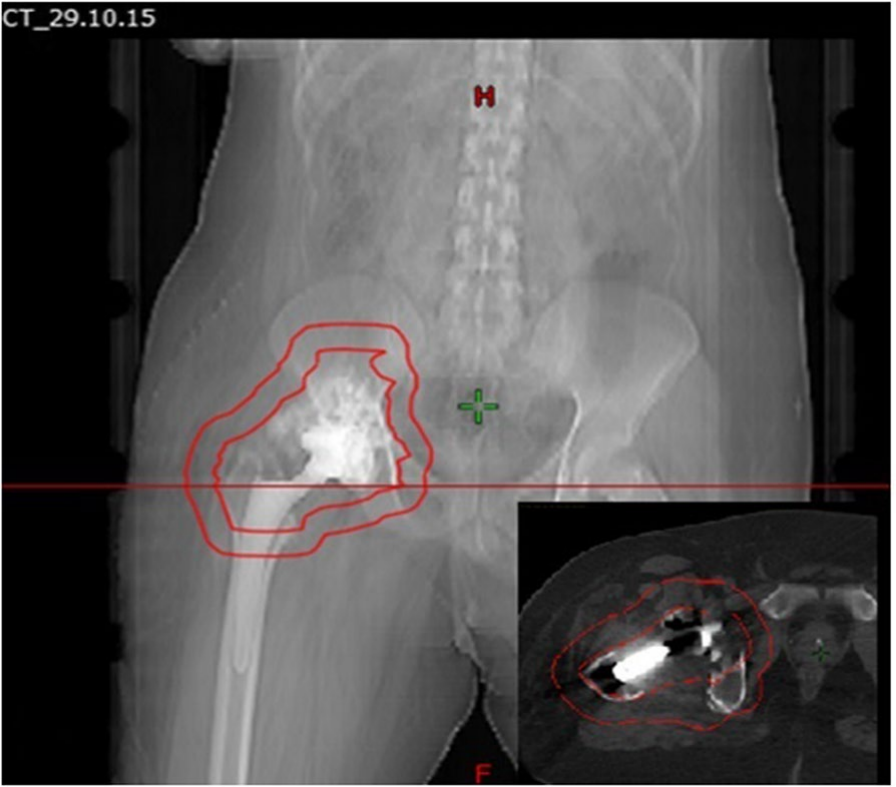
Hip heterotopic ossification contouring. Orthogonal view of contouring a patient with Rt hip heterotopic ossification performed total hip arthroplasty in addition to CT slice showing clinical target volume and planning target volume

The radiation is delivered within 24 h postoperatively. During CT simulation, the patient is evaluated by the radiation oncologist, radiation therapist, and physicist to choose the best comfortable and suitable treatment position. The selected position should enable testicular avoidance and/or shielding for male patients. CT images of 5-mm slice thickness were acquired, including the area of interest, using a large-bore CT simulator (Somatom, Siemens, Germany). Then, the physicists transfer the images by Eclipse (Varian Medical Systems, Palo Alto, USA) treatment planning system (TPS). The radiation oncologist delineates clinical target volume (CTV), which includes the periarticular tissues with the operative bed according to the affected joint. In hip joint HO, it is recommended that CTV encompass the area between the greater trochanter and ilium, the space between lesser trochanter and ischium, and the upper 1/3 of the artificial hip in cases of total hip arthroplasty (THA). Expansion of the CTV by 1 cm to create planning target volume (PTV) is performed to compensate for inter-and intra-fraction uncertainties (Fig. 2). The organs at risk are contoured, including the testis, ovaries, and small bowel, if applicable.

A dose of 7 to 8 Gy is prescribed to PTV, and a 3D-CRT or IMRT plan is created. The TPS is capable of homogeneity correction that compensates for artificial joints, plates, and screws. The plan is optimized so that at least PTV is covered by the 95% isodose line. To avoid permanent azoospermia, the mean testicular dose should be kept as low as possible and not exceed 1 Gy. The tolerance dose (TD) for ovaries is set at 4 Gy with more concern for the contralateral one. The prescribed radiation dose is delivered using the VARIAN Linear accelerator, series 2100C/D, Clinic DVHX. The electronic portal imaging device (EPID) is used to ensure accurate delivery of treatment. The absolute dose is verified for male patients using metal-oxide-semiconductor field-effect transistor (MOSFET)—a dose verification system [TN-RD-70-W], Best Medical Canada—as per physician request. The patients are followed up at the orthopedic clinic by X-ray or osseous CT every 3–6 months to exclude recurrence or malunion postoperatively during the first 2 years. Physiotherapy and occupational therapy are routinely continued per the patient clinical status [12–16].

### Follow-up and outcome assessment

The medical records were reviewed retrospectively using HIS. The patients who are not under regular follow-up at their hospital are contacted by phone for assessment and confirmation of data. The following variables were studied, age, gender, etiology, presenting symptoms, type of surgical resection, the prescribed radiation dose to the target volume (TV), dose to OAR, and treatment-related complications.

HO recurrence is defined radiologically as ossification progression after completion of radiotherapy by reviewing the series of reported X-rays on the picture archiving and communication system (PACS). The HO failure-free status is measured from the date of surgery till the date of the first reported HO recurrence.

Treatment failure is defined clinically or radiologically in the form of decreased ROM, non-union, refracture, or HO recurrence. The treatment failure-free status is measured from the surgery date until the reported failure.

The radiation-induced acute side effects were defined as any related complication that happened within 3 months from PORT. The radiation-induced chronic side effects were defined as any related complication that happened more than 3 months following treatment. The main studied late complications include the 2nd malignancy and infertility. The statistical package for social science version 21 (SPSS v21) was used for statistical analysis; we used the Kaplan-Meier method to estimate the recurrence-free rate.

## Results

A total of 127 HO patients were screened; 43 patients had an early stage, 26 patients had incomplete data, 12 patients had advanced disease with other reasons rather than MVA, and seven patients with a history of malignancy before the development of HO. Thirty-nine patients who had G4 Brooker’s classification for hip joint or Class IIIc Hastings and Graham classification for the elbow joint were included in the study. They all had a history of MVA and HO treated by surgical resection followed by PORT. Table 2 shows the patients’ characteristics.

**Table 2:** Patients’ characteristics and treatment outcome

Variable	No of patients (%)
Age (years)	13–47 (mean 27±8)
Sex	
Male	38 (97%)
Female	1 (3%)
Cause of HO*	
Traumatic fracture	27 (69%)
Brain injury	5 (13%)
Spinal cord injury	4 (10%)
Combined fracture and neurological injury	3 (8%)
Site	
Hip	35 (90%)
Elbow	4 (10%)
Referral hospital	
Our hospital	22 (56%)
Outside hospital	17 (44%)
Surgical resection	
Complete resection	3 (8%)
Partial resection	36 (92%)
Type of Surgical resection	
Joint replacement	7 (18%)
Surgical resection	32 (82%)
Radiotherapy given after	
1st resection	33 (85%)
2nd resection	6 (15%)
Dose of radiotherapy	
700 cGy	34 (87%)
800 cGy	5 (13%)
Testicular dose assessment	
Max calculated dose	81±83 cGy
Mean calculated dose	33±44 cGy
Measured dose (23 patients only)	58±40 cGy
Follow-up time (months)	24–144 (mean 74±33)
Time interval between trauma and surgery (months)	6–71 (mean 23±16)
Time interval between surgery and radiotherapy	
1 day	34 (87%)
≥ 2 days	5 (13%)
Median time to treatment failure (months)	3 (range 1–24)
Clinical status after treatment	
Regain full range of movement	18 (46%)
Regain partial range of movement	20 (51%)
No movement	1 (3%)
Clinical outcome at the time of the last follow-up	
No failure	33 (85%)
Treatment failure	6 (15%)
Treatment outcome	
2y-TFFR**	87.2±5.4%
8y-TFFR	79.3±9%
5y-HOFFR***	97.2±3%

HO* heterotopic ossification, TFFR** treatment failure-free rate, HOFFR*** heterotopic ossification failure-free rate

Most patients were males (97%) with a mean age of 27±8 years and a range from 13 to 47 years. Thirty-five patients had G IV Brooker’s classification hip joint HO, and four had Class IIIc Hastings and Graham elbow joint HO. All patients presented with complete loss of range of movement (ROM) of the affected joint, and only 17 out of 39 patients (44%) had pain. Twenty-seven patients (69%) had a traumatic fracture as a cause of HO. On the other hand, nine patients (23%) had brain and spinal cord injuries with long-standing bed-bound stays. Three patients had combined traumatic fractures and neurological insult.

The time interval between the MVA and resection of HO ranges from 6 to 71 months (mean 23±16). The complete surgical resection was performed for 8% of patients only, and 92% had partial resection with evidence of residual ossification in the surgical bed documented by postoperative imaging. Thirty-two out of 39 patients representing 82% had excision of existing HO with or without internal fixation and joint preservation, while seven patients had a joint replacement.

Thirty-three out of 39 patients (85%) received PORT after the 1st resection of HO, and six patients (15%) after the 2nd resection of HO. Thirty-four patients (87%) received radiation 24 h postoperatively, and all received a seven Gy single dose. Five patients (13%) received an 8 Gy single dose: two received radiation after 48 h, and three after 72 h postoperatively.

By reviewing TPS, the maximum testicular calculated dose was 81±83 cGy, and the mean was 33±44 cGy. Only 23 patients were found to have an absolute measured testicular dose as per HIS. The mean measured dose was 58±40 cGy. Only one female patient received radiotherapy to the hip joint with a mean calculated dose to the ipsilateral ovary of 210 cGy and the contralateral ovary of only five cGy.

The mean follow-up time was 74 months (24–144 months) with a median follow-up time of 66 months. Eighteen patients (46%) regain full ROM after treatment, and 20 (51%) regain partial ROM. One patient failed to regain active joint movement due to extensive nerve entrapment and affection; however, he benefited from passive joint movement and better posture. Table 2 shows the treatment outcome.

**Fig. 3:**
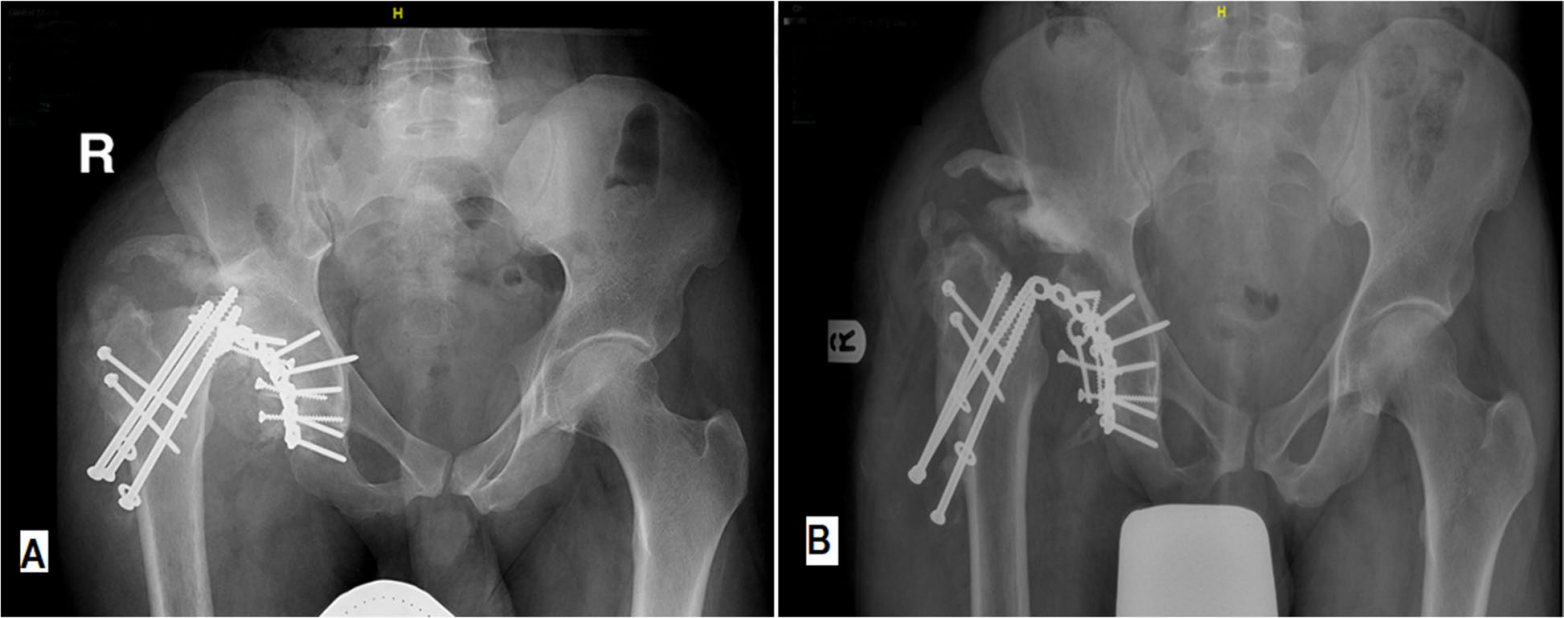
Patient with HO developed treatment failure postoperatively. (X-ray for the patient with Rt hip HO, **A** showed preoperative HO and B showed 3 months postoperative treatment failure)

At the time of the last follow-up, 33 patients had no treatment failure, while six patients proved to have treatment failure. The median time to failure was 3 months (ranging from 1 to 24 months). One patient failed due to recurrence of HO that warrants further surgical intervention with no additional radiation after re-excision of HO after 24 months. The other causes of treatment failure were non-union (2 patients), joint dislocation (1 patient), and combined refracture with joint dislocation (2 patients), as shown in (Fig. 3).

**Fig. 4:**
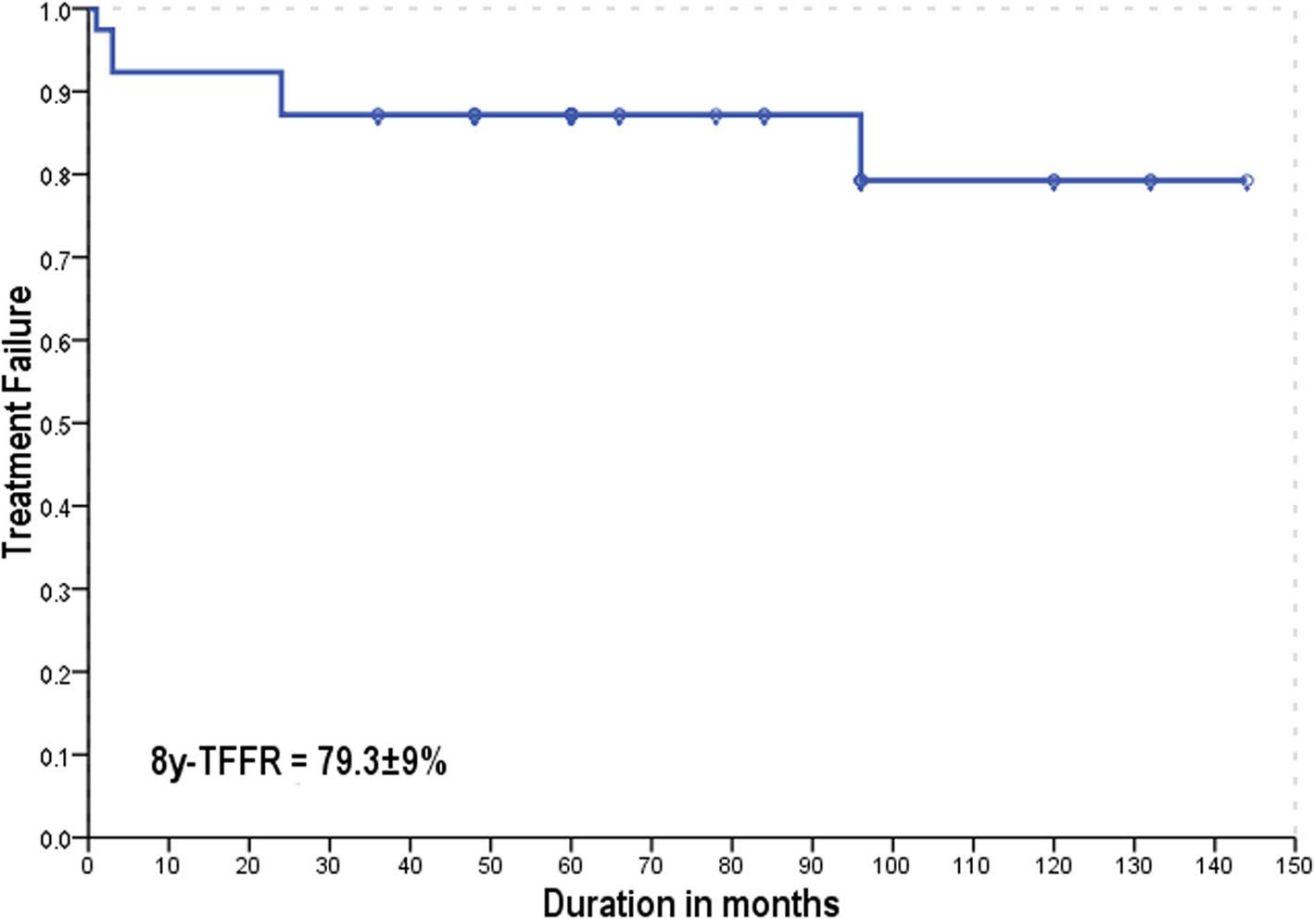
Treatment failure. (The curve shows the treatment failure-free rate {TTFS})

**Fig. 5:**
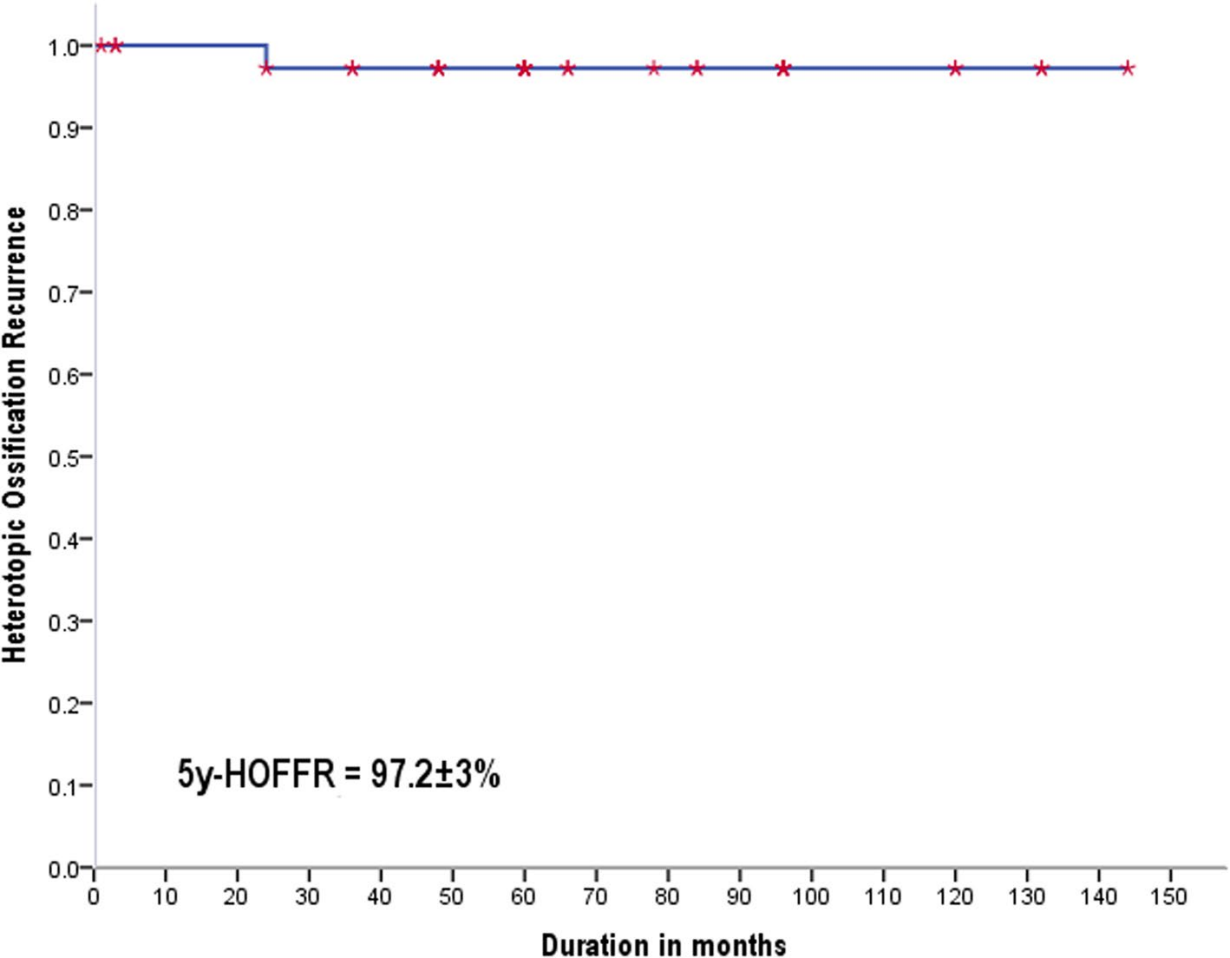
Heterotopic ossification recurrence. (The curve shows the heterotopic ossification failure-free rate {HOFFR})

The 2y-TFFR and 8y-TFFR (Table 2) were 87.2±5.4% and 79.3±9%, respectively (Fig. 4); however, the 5-year HO failure-free rate (5y-HOFFR) was 97.2±3% (Fig 5). The time interval between surgical resection and treatment failure rather than HO recurrence ranges between 1 and 3 months indicating surgical reasons.

A total of 5 patients (13%) experienced acute complications; 3 out of them developed gastrointestinal symptoms in the form of diarrhea and abdominal colic, while two patients developed mild skin reactions. Acute side effects were resolved without any consequences. There is no reported postoperative infection in our patients; however, this could be related to routine prophylactic antibiotic administration postoperatively.

Despite a relatively long follow-up time, we did not report any late complications like infertility or secondary malignancies related to the radiation area. One patient developed thyroid cancer 9 years after radiation treatment which was entirely outside the irradiated area.

Infertility as a late complication was not reported or correlated to radiation treatment. If we excluded the elbow HO and the female patient, we had 23 measures for the testicular dose out of 34 patients, with all measurements indicating safe radiation exposure. The only lady included in the study delivered healthy babies after radiation treatment. Four males out of the 34 patients who received radiation to the pelvis did not marry because of their young age and were not tested for infertility. Five of the 12 patients with brain or spinal cord injuries were bedridden and did not marry. All of the previously mentioned nine male patients did not have the potential to do semen analysis at the research time because of age and general condition. The other 25 patients out of the 34 patients got kids post-radiation therapy. Four of the 25 patients were under treatment for oligospermia but succeeded in having offspring. The testicular measured doses for the four patients were available and less than 50cGy.

## Discussion

Our retrospective study included patients having a history of MVA with major fractures and/or neurological injuries. Those patients presented with G4 hip joint HO and class IIIc elbow HO. The reason for studying this cohort of patients is to highlight the importance of PORT as an option to reduce local recurrence. In a study of 410 HO patients by Seegenschmiedt et al., those who presented by G3-4 Brookers’ classification had the highest failure rate following resection of HO; 39% compared to 7% for G1-2 HO patients with *p* value < 0.001 [17].

Our patients received 7 to 8 Gy single-dose postoperatively, which matches the standard practice of many centers worldwide. The use of such a low dose has been justified by Craven et al. since 1971. He proved that target cells for RT are the radiosensitive osteogenic progenitors that help transform primitive mesenchymal cells in the surrounding soft tissues into the osteoblastic tissue and mature lamellar bone (14). The before-mentioned cause justifies the need for early delivery of PORT by targeting the most sensitive progenitor cell before maturation to the more radioresistant osteogenic cells.

Many authors clinically studied the preoperative versus postoperative radiation and single versus fractionated dose. The results were contradictory, with more belief in the postoperative single-shot strategy among radiation oncologists. Gregorich et al. randomized 122 patients to preoperative versus postoperative radiation using a single dose of 7–8 Gy. Out of 98 evaluable patients with a median follow-up of 38 weeks, no significant differences exist. The HO treatment failure rate was 26% for preoperative vs. 28% for postoperative (*p* value = 1.0). The reported G3-4 Brookers’ classification recurrence was 2% for preoperative patients vs. 5% for postoperative patients with *p* value = 0.58 [18]. However, Seegenschmiedt reported 5% radiological failures in the postoperative compared to 19% in the preoperative group with *p*<0.05 [17]. Healy et al. also stated that lowering the single dose below 7 Gy increases the failure rates. He compared 7 Gy and 5.5 Gy postoperatively in 107 hips. Heterotopic ossification developed in 63% of patients treated with 5.5 Gy compared to 10% of patients treated with 7 Gy. The difference was statistically significant [19]. Pellegrini et al. compared an 8-Gy single dose with a fractionated dose of 10 Gy in 5 fractions in 62 patients and reported a 21% failure rate equivalent in both groups [20]. Two meta-analyses published by Popovich and Milakovic Popovic studied 5464 treatments retrospectively, and Milakovic performed a meta-analysis of 12 well-designed controlled randomized trials. Both metanalyses recommended the proposed dose of 7–8 Gy either preoperatively or postoperatively [21, 22].

The studied patients received PORT within 3 days of surgery; however, 87% received the radiation within 24 h. Considering that 44% of our patients were referred from another hospital, the 13% reported delay is justified. To our knowledge, this point is not well studied previously in randomized trials. Most published studies recommend radiation delivery within 4 days. Freije et al. reported an increased risk of HO recurrence with delayed PORT following surgery with a marginal statistical significance of *p* value equal to 0.0559 [23]. We did not study this point due to the limited number of patients who received delayed treatment in our cohort of patients. Interestingly, Marcos reported that delayed PORT could stop the progression of HO recurrence in 89% of patients. The study included only nine patients who received late PORT 6 to 12 weeks following surgery once early recurrence was observed radiologically [24].

To compare our data with others, we should highlight the long-time interval between trauma and surgical resection of HO (mean=23 months) besides the advanced stage of our patients. The prolonged delay between trauma and resection of HO in cases with ankylosis of the hip, epiphyseal osteoporosis, and cartilage loss may occur, causing joint fusion and hence high liability of surgical failure. The HO recurrence was evident in only 2.5% of our patients, while 12.5% had non-union, refracture, and joint dislocation with a mean time for treatment failure of 3 months. Seegenschmiedt et al. reported 5% radiological failure in 81 patients randomly assigned for PORT among 161 patients studied [17]. Also, Freije et al. retrospectively analyzed 287 patients, with 12 (4.2%) who experienced HO recurrence [23]. Noting that, only 1.4% of Freije patients developed G3-4 Brooker’s HO recurrence that warrants further surgery. The median time to treatment failure was 8.6 months (2.8–24.5 months) compared to 3 months (range from 1–24 months) in our cohort of patients. Also, Balboni et al. reported treatment failure in the form of non-union in 12–30% after RT and 2–15% for those who did not get RT [25]. This data is comparable to our 12.5% non-union and joint dislocation data, as mentioned before.

The 3D-CRT and IMRT—routinely used in our department—are proven to decrease HO recurrence after PORT, as stated by Mourad et al. [26]. In a retrospective study of 532 patients, the overall failure rate was 21.6% but only 6.6% among those who underwent 3D-CRT. Again, this is comparable to the 2.5% recurrence rate in our present study. On the other hand, Burnet showed a higher failure rate than ours. Seventeen percent of 34 studied patients developed G 1 Brooker’s HO recurrence [27].

We attributed our study’s 12.5% treatment failures to advanced disease presentation and delayed interference following trauma. Despite the previously highlighted challenges faced by orthopedic surgeons in addition to disease patterns, our results are comparable to most published data.

Our reported acute side effects were tolerable, self-limiting, and did not affect the treatment outcome or quality of life. We did not report in-field second malignancy but only 1 case of thyroid cancer outside the radiation field. Our second malignancy results are consistent with Freije et al. published data of 287 patients, as they did not report any in-field 2nd malignancy but two 2nd malignancies outside the radiation fields [23].

Similarly, all large HO studies denied the second malignancy as a late complication and just warning from that serious complication, but there are 2 case reports published of significant importance. Mourad et al. reported high-grade undifferentiated sarcoma of the femur post two courses of PORT for recurrent HO after 16 months of his second course [28]. Farris et al. also reported osteosarcoma of the pelvic bones in a 26-year-old male 11 years after 7 Gy of radiotherapy [29]. The last case at least received non-conformal radiation, which is not the standard currently. This patient was treated using an open 8 × 15 anteroposterior-posteroanterior (AP/PA) 6-MV photons to 7 Gy in one fraction, without bone shielding.

Regarding the testicular dose of our studied patients, the mean measured dose was 58±40 cGy. As expected, this reading is more than the TPS mean calculated dose, which was 33±44 cGy, due to scattering dose issues. These data were assuring for fertility preservation in our studied patients. Patel et al. highlighted fertility problems as a potential side effect of radiotherapy in young males. They recommended keeping the RT doses in the range of 70 to 100 cGy to avoid permanent azoospermia with every attempt to be made for testis shielding [30]. We did not report any significant infertility problems among our studied patients. Only four patients were found to have temporary infertility and were under follow-up in the infertility clinic. All of them had succeeded in getting children after radiation.

Many studies have demonstrated local and systemic altered levels of inflammatory cytokines associated with HO formation. The high serum levels of TNFa, IL-1b, IL-6, and MCP-1 were noticed with HO formation in animals, as shown by Sung et al. [31]. The positive correlation between increased inflammatory cytokines and HO formation opens a promising research field for diagnosing high-risk patients among those with recurrent HO. Also, this will open a new frontier for treatment options [32, 33].

The study of a particular pattern of patients—G4 Brooker’s classification hip HO and class IIIc Hastings and Graham classification elbow HO patients following MVA—is a strong point of our study compared to other studies. Also, the long follow-up time of the studied patients valued our safety results. On the other hand, the retrospective study and the relatively small number of patients are considered the main limitations of our research.

## Conclusions

Our focused study on the most advanced-stage HO patients who had surgical resection confirmed the efficacy of PORT in preventing HO relapse. With the long follow-up time, we can state that the risk of radiation-induced second malignancy and absolute infertility is negligible, especially with 3D-CRT and IMRT. However, we recommend testicular dose measurement and adjustment to ensure radiation safety and avoid infertility. The leading cause of failure was surgical can be attributed to the advanced presentation. We recommend further studies to test the recently defined markers associated with the development of HO to optimize the role of RT for HO patients and high-risk patients—following major surgeries—vulnerable to HO development.

## Data Availability

Research data are stored in our institutional repository and will be shared upon request with the corresponding author.

## Abbreviations

HO: Heterotopic ossification
PORT: Postoperative radiotherapy
MVA: Motor vehicle accidents
TFFR: Treatment failure-free survival
HOFFR: Heterotopic ossification failure-free survival
ROM: Range of movements
RT: Radiotherapy
3D-CRT: 3-Dimensional conformal radiation therapy
IMRT: Intensity-modulated radiotherapy
OAR: Organs at risk
MOH: Ministry of Health
IRB: Institutional review board
HIS: Hospital information system
TPS: Treatment planning system
CTV: Clinical target volume
THA: Total hip arthroplasty
PTV: Planning target volume
TD: Tolerance dose
EPID: Electronic portal imaging device
MOSFET: Metal-oxide-semiconductor field-effect transistor
TV: Target volume
PACS: Picture archiving and communication system
SPSS: Statistical package of social sciences
